# The Switch from Low-Pressure Sodium to Light Emitting Diodes Does Not Affect Bat Activity at Street Lights

**DOI:** 10.1371/journal.pone.0150884

**Published:** 2016-03-23

**Authors:** Elizabeth G. Rowse, Stephen Harris, Gareth Jones

**Affiliations:** School of Biological Sciences, Life Sciences Building, University of Bristol, 24 Tyndall Avenue, Bristol, BS8 1TQ, United Kingdom; University of Western Ontario, CANADA

## Abstract

We used a before-after-control-impact paired design to examine the effects of a switch from low-pressure sodium (LPS) to light emitting diode (LED) street lights on bat activity at twelve sites across southern England. LED lights produce broad spectrum ‘white’ light compared to LPS street lights that emit narrow spectrum, orange light. These spectral differences could influence the abundance of insects at street lights and thereby the activity of the bats that prey on them. Most of the bats flying around the LPS lights were aerial-hawking species, and the species composition of bats remained the same after the switch-over to LED. We found that the switch-over from LPS to LED street lights did not affect the activity (number of bat passes), or the proportion of passes containing feeding buzzes, of those bat species typically found in close proximity to street lights in suburban environments in Britain. This is encouraging from a conservation perspective as many existing street lights are being, or have been, switched to LED before the ecological consequences have been assessed. However, lighting of all spectra studied to date generally has a negative impact on several slow-flying bat species, and LED lights are rarely frequented by these ‘light-intolerant’ bat species.

## Introduction

Increased use of artificial lighting over the last century has resulted in extensive changes in the nocturnal landscape [1,2]. Although artificial lighting benefits people [3,4], light pollution is widespread [5,6] and can affect organisms across a range of spatial scales [7].

**Fig 1:**
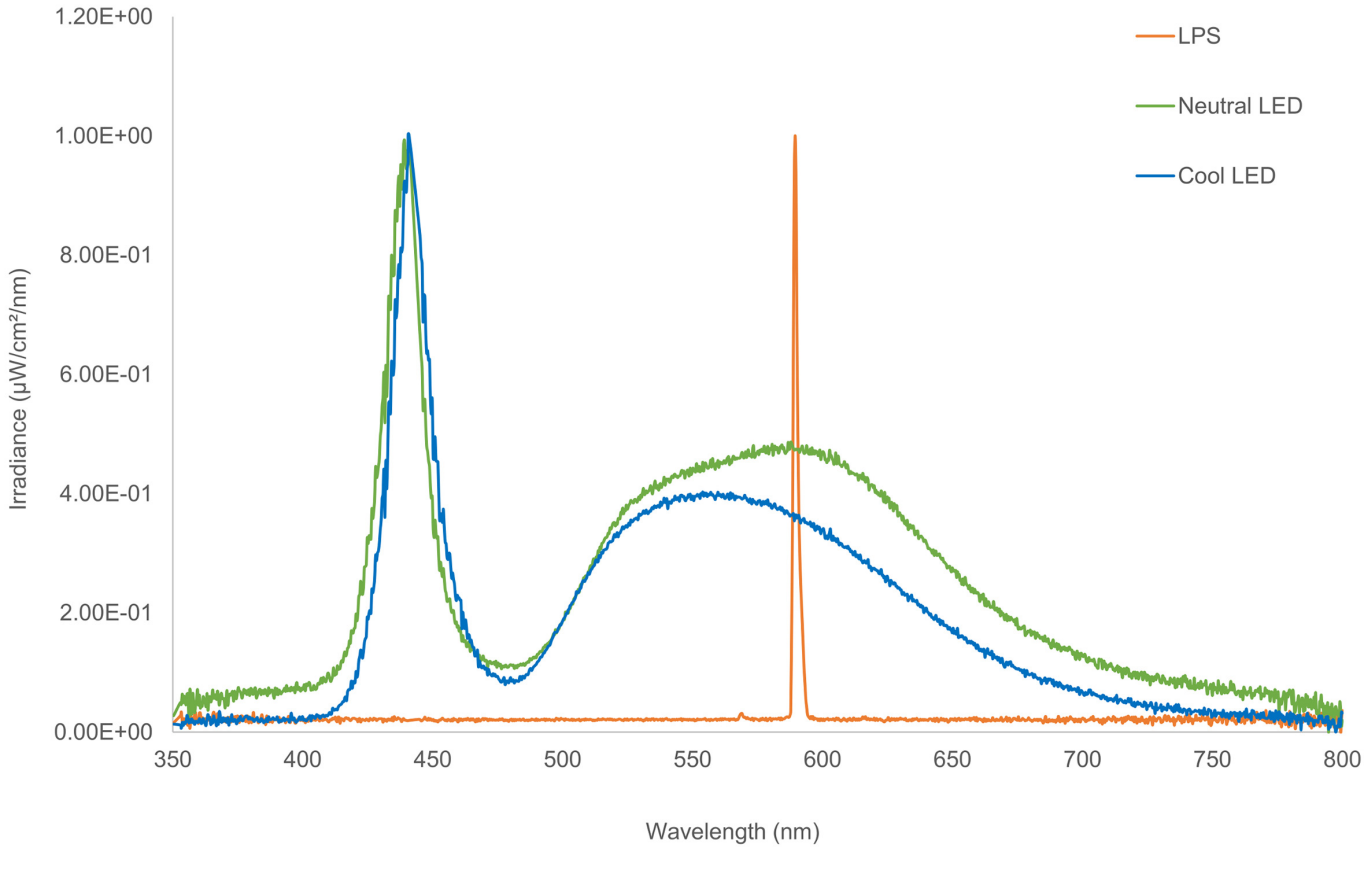
The spectral output of LPS and LED street lights, representative of the lights used in this study. LPS and neutral LED spectral outputs were taken from site J and the cool LED spectral output from site G, shown in Fig 2.

Street lights are widely used around the world and have the potential for far-reaching effects on the environment, biodiversity and human health [8,9]. During the first part of the 21^st^ century, the number of street lights in the UK continued to increase by 3% per annum [5] and their spectral signatures, i.e. the range of wavelengths that the lights emit, have changed [10,11]. There is currently a shift in street lighting from narrow light spectrum sources such as orange low-pressure sodium (LPS) and yellow high pressure sodium (HPS) lights to broad spectrum “white” lighting technologies such as light emitting diodes (LEDs) [9,12,13] (Fig 1). There are three types of LED lights, cool, neutral and warm, that vary according to their correlated colour temperature (Kelvins). Cool LEDs appear ‘cold’ and have a high colour temperature (~6000 K), warm LEDs have a ‘warmer’ appearance (~2700 K), and neutral LED lights have a colour temperature between cool and warm LED lights (~4000 K) [14]. LED lights have a number of advantages, including increased energy efficiency, directionality, controllability (ability to dim and switch-off when not in use), longevity and flexibility of colour choice [9,13,14]. LED lights also have a higher colour rendering index (CRI), which expresses the capacity for a light source to yield the “true” colour of an object in relation to human vision [14]. Street lights exist primarily for perceived human safety benefits, and improved colour rendering for human vision enables people to see their surroundings more clearly, making them feel less vulnerable at night [15].

While these changes in spectral output accommodate human vision, many organisms have different spectral sensitivities [16]. Insects, for instance, are attracted to shorter wavelengths, particularly near the UV part of the spectrum, as this corresponds with the peak spectral sensitivities of their eyes [17–19]. Hence insects are common around old technology gas discharge street lamps that contain a high proportion of short wavelengths, such as high pressure mercury vapour (HPMV) lights [18]. However insects are rarer around LPS lights, which are essentially a monochromatic light source [20].

Bats are a valuable taxon for understanding some of the ecological impacts of artificial light since they exhibit species-specific responses to lighting; some bat species feed on insects attracted to street lights, whereas others avoid light [21,22]. Street lights attract fast-flying bats such as those in the genera *Eptesicus*, *Lasiurus*, *Nyctalus* and *Pipistrellus*, most probably because they prey on the insects attracted to the street lights [23–25]. These bats share a number of traits including aerial hawking [26], foraging in open habitats [27] and emerging relatively early after sunset, which is believed to coincide with peak insect availability [26]. *Eptesicus* and *Nyctalus* species tend to fly above street lights, diving near the light cone to feed, whereas *Pipistrellus* species hunt in and out of the light cone [12,28]. *P*. *pipistrellus* bats spend the majority of their time in dim or dark areas [29,30], so are only likely to use lights if the benefits associated with increased foraging success outweigh the perceived risk of predation [25]. In contrast, slow-flying bats such as *Myotis*, *Plecotus* and *Rhinolophus* species do not appear to be attracted to artificial lights [21,25,31]; these species rarely feed around street lights, possibly because the perceived risk of predation may be too high [26,32]. If artificial lights dominate the landscape, it may greatly reduce the high quality habitat available to these slow-flying species. A major concern is that the spread of artificial lights will have long-term effects on these slow-flying, light-intolerant bats.

Many local authorities across Britain are in the process of switching their old LPS and HPS lights to LED lights. One of the main drivers is cost, as local authorities can save money from reduced energy use and maintenance costs. Similar changes are happening across continental Europe and elsewhere in the world. However, LED lights are spectrally different from either LPS or HPS lights, the predominant street lights in the UK and around the world [33]. Species have existed under the yellow and orange hues emitted by sodium street light for decades; how bat activity will change following the introduction of modern broad spectrum lights is unclear [22]. The effect of an artificial light on each organism will depend on its photoreceptors, the spectral output of the light source, the intensity of the light and reflectance from the surrounding environment [9]. With that in mind we investigated how the switch-over from LPS to LED street lights affected bat activity and feeding behaviour.

## Methods

### Ethics Statement

All the data were collected remotely and there was no animal handling or manipulation. The study was reviewed and approved by the University of Bristol Ethics Committee–approval number UB/14/031.

### Site description and experimental set-up

**Fig 2:**
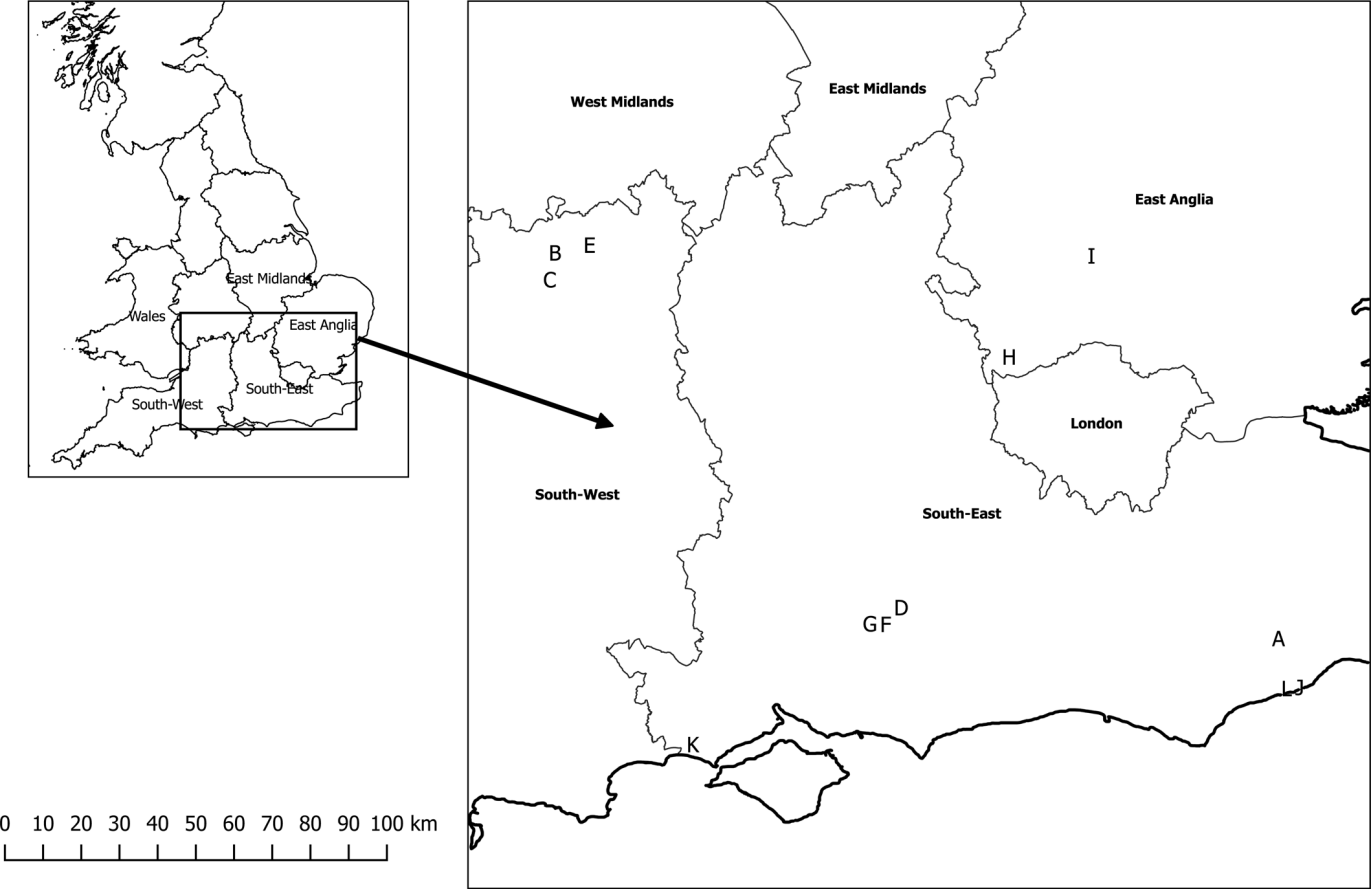
Locations of the 12 study sites in southern England. There were two lighting columns (street lights) at each site, one control, the other experimental.

A before-after-control-impact paired design (BACIP) [34], based on a previous study [35], was used to examine the effects of a switch from LPS to LED street lights at twelve sites in four counties (East Sussex, Gloucestershire, Hampshire and Hertfordshire) across southern England (Fig 2). A BACIP identifies if the impact being tested affects the system in question as it controls for variables such as environmental factors and seasonal changes [36], and so it was essential that the control and experimental lighting columns were matched as closely as possible. We used existing street lights and so site choice was governed by where local authorities were switching from LPS to LED street lights. Each site consisted of a pair of lighting columns (street lights), one control (remaining LPS throughout the study) and one experimental (changing from LPS to LED). Control columns were restricted to areas where LPS lights remained the dominant street lights throughout the study, whereas experimental columns were restricted to areas where LPS lights were the dominant lighting type before switch-over and LED lighting after switch-over. Paired columns were separated by a mean distance of 1.4 km (s.d. ± 0.9 km) to reduce the chance of recording the same bats around the control and experimental lighting columns. Sites were separated by a minimum distance of 1.86 km to ensure the samples were independent.

The sites were in suburban areas close to bat commuting and foraging habitats [37]; ten sites were in residential areas, the other two (sites H and I) were on A-class roads. Aerial imagery on Google Earth was used to match the distance to wooded areas, freshwater and grassland as closely as possible between the control and experimental columns (Table 1), although some variation was inevitable because this was a “real-life” experimental set-up. However, all sites were no greater than 154 m from a wooded area, defined as a stand of >10 continuous trees (mean 54.0 m, s.d. ± 32.3 m), 709 m from a freshwater source (mean 220.0 m, s.d. ± 182.2 m) and 428 m from grassland with an area >0.6 ha (mean 110.9 m, s.d. ± 110.1 m). Within sites, there was a mean difference between the control and experimental columns of 35.4 m (s.d. ± 32.5 m) between distance to a wooded area, 130.4 m (s.d. ± 144.5 m) to a freshwater source and 63.0 m (s.d. ± 50.7 m) to grassland.

**Table 1:** Specifications of the LPS and LED street lights used in this study. Control and experimental lighting columns in each pair were matched in terms of height (m) and output (watts) prior to switch-over. For the experimental columns, the output and illuminance readings of the LED lights after switch-over are shown in brackets. Proximity to key habitats is shown for each column. Letters denote the 12 study sites; the location of each site is shown in Fig 2. Power (watts) can reduce, but illuminance (lux) can stay the same or increase after the switch-over to LED lights. This is because LED lights are more energy efficient and the lanterns are more directional than LPS lights.

Site	Column	Column height (m)	Power (watts)	Illuminance (lux)	Distance to a wooded area (m)	Distance to freshwater (m)	Distance to grassland (m)
A	Control	6	55	41	22	71	339
A	Experimental	5	35 (14)	28 (28)	56	258	161
B	Control	5	35	28	45	515	51
B	Experimental	5	35 (19)	31 (71)	91	465	28
C	Control	5	35	3	55	55	67
C	Experimental	5	35 (27)	3 (57)	78	413	25
D	Control	5	26	8	60	390	131
D	Experimental	5	26 (10)	3 (20)	52	333	46
E	Control	5	35	36	36	29	64
E	Experimental	5	35 (27)	31 (60)	154	161	9
F	Control	5	26	28	46	53	58
F	Experimental	5	26 (10)	41 (20)	54	90	135
G	Control	5	26	2	81	59	30
G	Experimental	5	26 (10)	3 (19)	91	59	82
H	Control	10	91	60	87	215	17
H	Experimental	10	91 (107)	60 (109)	17	397	16
I	Control	10	91	118	7	14	55
I	Experimental	10	91 (107)	114 (178)	59	68	2
J	Control	5	35	29	31	206	127
J	Experimental	5	35 (14)	40 (29)	4	175	177
K	Control	5	26	2	50	252	163
K	Experimental	5	26 (10)	28 (18)	33	709	156
L	Control	5	35	41	38	137	295
L	Experimental	5	35 (14)	40 (25)	50	157	428

While there was variation between sites, control and experimental columns in each pair were matched for height (m), output (watts) and illuminance (lux). The local authorities provided information on the light type, output and column height. Within sites, the column heights, light type and output were identical between the control and experimental columns except for site A, where the control column was 6 m in height and had an output of 55 watts, whereas the experimental column was 5 m in height and had an output of 35 watts (Table 1). A combination of neutral and cool LED lights (4000-5700K) was used. Light measurements from control and experimental lighting columns were taken with a lux meter (photometric system) and a spectrometer (radiometric system) to ensure that the light output and intensity of the paired street lights were comparable. Illuminance was measured with a TES 1330 lux meter (ATP Instrumentation Ltd, Leicestershire, UK) held horizontally 1.8 m from the ground directly beneath the street light. Irradiance (absolute intensity of the street light) was measured (μW/cm^2^/nm) 4 m directly below the lantern with a tripod and using a calibrated Ocean Optics USB 2000 spectrometer, a P200-5-UV/VIS patch cord and a CC-3 cosine corrector. A Gershun tube was used to reduce the acceptance angle (the amount of light that falls on the sensor) to ensure that the irradiance measurement was from the street light. Ensuring all light readings were taken 4 m from the lantern enabled absolute intensities to be compared between columns of varying heights. Since environmental variables such as temperature, precipitation and cloud cover affect light readings [38], we took light measurements on clear dry nights when there was no full moon.

### Measuring and identifying bat calls

Field work took place between May and October 2014. Bat activity was measured using Song Meter SM3 Bat Recorders (Wildlife Acoustics Inc., Massachusetts, USA). Prior to deployment, all detectors were tested in a semi-anechoic chamber and the microphone placed 1 m and at an angle of 45° from the speaker of an ultrasound generator, which then played a series of high frequency sounds between 20 and 120 kHz. All detector systems used were comparable in sensitivity as determined by visual inspection of waveforms in BatSound (Pettersson Elektronik, Uppsala Science Park, Sweden). Four detectors were used to further minimise bias: they were randomised between sites, but the same detector was used before and after switch-over for both the control and experimental lighting columns.

Street sign and tamtorque sign fixing clamps were used to attach the bat detectors on average 1.09 m (range 0.73 m to 2.07 m) from the lantern to ensure a standardised method across lighting columns (S1 Fig). Recordings were made simultaneously at both the control and experimental columns for three consecutive nights before and after the switch-over. Bat detectors were set to record bat activity using triggers from thirty minutes before sunset on the first night until thirty minutes after sunrise on the fourth morning. The microphone on the detector was pointing in the same direction as the lantern. All detectors ran the same program, which was generated on SM3 Configurator 1.2.4 (Wildlife Acoustics Inc., 2015) and files were stored as waveform audio files (WAV). The settings on the detectors were: high pass filter 16 kHz; sample frequency 384 kHz; minimum frequency 16 kHz; maximum frequency 120 kHz; maximum recording time 15 seconds; and trigger level 12 dB. Detectors were removed between treatments and post switch-over recordings were made a minimum of seven days (mean 14.9 days, s.d. ± 5.3 days) after conversion to enable the bats to adjust to the new lights [35].

It is not possible to record individual bats using acoustical methods, so bat activity was monitored as the number of passes over the three recording nights. A bat pass was defined as when the time between pulse intervals was four times the interpulse interval [21,31,39]. We also investigated bat feeding behaviour around the control and experimental columns. Before catching an insect, a bat produces a feeding buzz, which is distinguishable from other echolocation calls by its higher repetition rate [32,40]. Relative feeding activity was measured using a ‘buzz ratio’, which is the proportion of call sequences that included feeding buzzes over the three recording nights [41]. Buzz ratio acted as a proxy for insect activity, the assumption being that the higher the buzz ratio, the more attractive the light source was to insects. We used buzz ratios as a measure of feeding relative to general activity at LPS and LED street lights.

We analysed the bat calls using the automatic identification software programme Kaleidoscope Pro (v0.1.1.20, Wildlife Acoustics Inc., Massachusetts, USA) with British Bat Classifiers (v1.0.5). All bat calls were also validated manually using Kaleidoscope viewer and Bat Sound with the parameter values stipulated in [42] to ensure correct identification. If there were any discrepancies between the manual and automatic methods of species identification, the manual identification was used. Manual validation was used to record multiple passes and/or species per file. Bats were identified to either species (*Eptesicus serotinus*, *Pipistrellus nathusii*, *P*. *pipistrellus* and *P*. *pygmaeus*) or species groups (*Myotis* spp., *Nyctalus* spp. and *Plecotus* spp.) depending on how diagnostic the calls of particular species were [43].

### Data analysis

The pairings were an integral part of the experimental design as they accounted for any environmental and/or seasonal changes between the two recording periods. To determine if the switch-over from LPS to LED street lights affected bat activity, we were interested in the difference in the number of bat passes before and after the switch-over between the control and experimental lighting columns [44]. If the LED lights did not affect bat activity, the difference between the control and experimental column in each pair would be negligible or inconsistent between pairs [45].

As the bat activity data were not normally distributed, we used a series of Wilcoxon signed rank tests to determine if there was a difference in the number of bat passes between LPS and LED street lights compared with differences in the paired control lights where no switch-over occurred. We compared bat activity of all species combined, and separately for *P*. *pipistrellus*, *P*. *pygmaeus* and *Nyctalus* spp., which together contributed 90% of all recorded bat calls. Similarly, buzz ratio data were not normally distributed and so a Wilcoxon signed rank test was used to test for differences between LPS and LED lighting columns. The buzz ratios of all species were compared, as were the data for *P*. *pipistrellus*, which contributed 80% of all buzz ratios recorded. Bonferroni corrections were used to adjust for multiple testing to reduce the risk of false positives; a significant difference between LPS and LED for the bat activity and buzz ratios was accepted if *p* < 0.0125 and *p* < 0.025 respectively [46]. Species richness and species diversity indices [47] were calculated to compare relative abundances of bat species around LPS and LED street lights; the diversity indices were based on the total number of bat passes for each species at control and experimental columns both before and after the switch to LED. All statistical and descriptive analyses were carried out in R Studio (version 0.99.451) [48]. The Wilcoxon signed rank test was conducted using the ‘coin’ package [49] and the species richness and species diversity indices were conducted using the ‘vegan’ package [50].

## Results

### Bat activity

There were 30,416 files from the 12 sites (24 columns). These contained 37,124 bat passes, an average of 1.2 passes per file: 70.0% of passes were *P*. *pipistrellus*, 13.0% *Nyctalus* spp., 9.4% *P*. *pygmaeus* and 7.7% *Myotis* spp. (electronic supplementary material, S1–S4 Tables). However, nearly all the *Myotis* spp. calls were recorded from site E after the experimental column had been switched to an LED light. *P*. *pipistrellus* were found at both control and experimental lighting columns across all sites, *P*. *pygmaeus* were found across all sites but only at ten of the twelve control columns, and *Nyctalus* spp. were recorded at all control lighting columns but only nine of the experimental columns.

**Fig 3:**
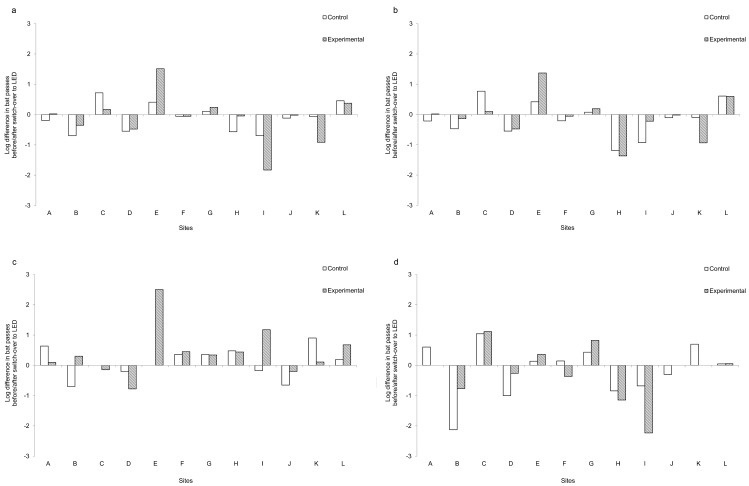
The differences in the log bat passes (number of bat passes after the switch-over minus the number of bat passes before the switch-over) for the control and experimental columns in each pair. A positive value indicates that there were more bat passes after the switch-over compared with before, and a negative value indicates more bat passes before compared with after the switch-over. (a) total bat activity, (b) *Pipistrellus pipistrellus*, (c) *Pipistrellus pygmaeus* and (d) *Nyctalus* spp. Letters denote the 12 study sites; the location of each site is shown in Fig 2.

There was no significant difference in the number of passes from all species before and after the switch-over to LED between the control and experimental columns (*W* = 30, *Z* = -0.706, *p* = 0.4802; Fig 3A). Bat activity was not significantly different between LPS and LED street lights for *P*. *pipistrellus* (*W* = 30, *Z* = -0.706, *p* = 0.4808; Fig 3B), *P*. *pygmaeus* (*W* = 36.5, *Z* = -0.1963, *p* = 0.8444; Fig 3C) or *Nyctalus* spp. (*W* = 35.5, *Z* = -0.2751, *p* = 0.7832.8136; Fig 3D). Thus the switch-over from LPS to LED street lights did not have a significant effect on either total bat activity or individual species/groups for which we had adequate data. In many cases the responses at the control and experimental lighting columns mirrored each other, i.e. when there was an increase in the number of bat passes at the control column, there was a similar increase at the experimental column and *vice versa*. Furthermore, the direction of change between the control and experimental sites was consistent across eleven of the twelve sites, although at sites E and I the magnitude of change at the experimental columns was greater than that at the control columns.

### Buzz ratios

**Fig 4:**
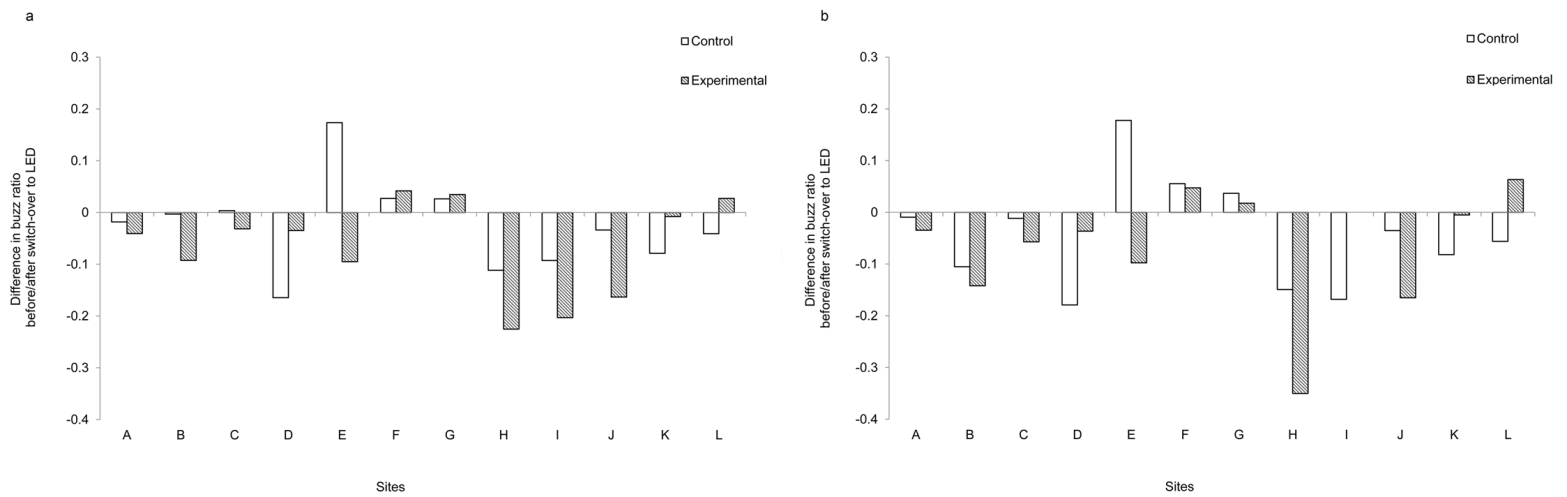
The difference in the buzz ratios (proportion of feeding buzzes after the switch-over minus the proportion of feeding buzzes before the switch-over) for both the control and experimental lighting columns. (a) all bat species and (b) *Pipistrellus pipistrellus*. Letters denote the 12 study sites; the location of each site is shown in Fig 2.

There was no significant difference in buzz ratios between the LPS and LED street lights for all bat species (*W* = 53, *Z* = 1.0983*p* = 0.2721; Fig 4A) or for *P*. *pipistrellus* (*W* = 46, *Z* = 0.5491, *p* = 0.5829; Fig 4B). As with the total number of bat passes, patterns of change at each site were usually the same at both the control and experimental columns. However, there was a marked difference at site E, where there was a decrease in the buzz ratio even though the number of bat passes at the experimental site increased by more than 32 times after the switch-over to a LED light (Fig 3A).

### Species richness

Species richness and diversity indices showed that the same species were in the vicinity of the LPS and LED lights and diversities remained consistent across the control and experimental sites for the two time periods (Table 2); thus the proportion of calls per species varied little between recording periods or light type.

**Table 2:** Species richness and Shannon-Wiener, Simpson’s and Fisher’s alpha diversity indices before and after switch-over from LPS to LED lights.

Sites	Species richness	Shannon-Wiener index	Simpson’s index	Fisher’s alpha index
Control -before	8	0.926	0.514	0.892
Control—after	8	0.677	0.351	0.939
Experimental—before	8	0.806	0.452	0.877
Experimental—after	7	0.906	0.496	0.691

## Discussion

The activity of all bats combined, and *Pipistrellus* and *Nyctalus* species, was not significantly different around LED and LPS street lights. While several studies have recorded fewer bats around LPS compared to ‘white’ street lights [23,24,25], the ‘white’ lights used in those studies were HPMV street lights that, unlike LED lights, emit UV light. Compared to other spectral emissions, UV light is attractive to many insects that bats prey on. Although LED lights contain more short wavelengths than LPS lights, it is likely that both light types are equally attractive to insects since neither contains UV light [24,26,35]. Moreover, we found that the buzz ratio did not change between the two types of street lights, again suggesting that LPS and LED lights had a similar effect on overall insect activity.

In addition to LED lights, there has been interest in the ecological impacts of other new lighting technologies such as metal halide lights. Both metal halide and LED lights are broad spectrum lights and so have a high colour rendering index [35] but, unlike LEDs, metal halide lights are a gas discharge lamp and emit a high proportion of short wavelengths, including some UV emissions [14]. Some local authorities are replacing LPS lights with metal halide rather than LED lights [51] to save money on installation costs. Unlike our study, activity of both *Nyctalus* and *Pipistrellus* species increased around metal halide compared with LPS lights in a BACIP experiment with fewer study sites [35]. However, while it was predicted that insect activity would be greater around the metal halide lights, the buzz ratio did not vary between the two light types, suggesting that the bats may be attracted to metal halide lights for some reason other than feeding. This may be related to the spectral sensitivities of bat eyes, as vesper bats can see UV light [52]. Alternatively, this may be due to the limitations of the AnaBat detectors (SD1 and AnaBat II; Titley Electronics, Ballina, New South Wales, Australia) used in that study [35]; these are less sensitive than the full spectrum Song Meter SM3 Bat Recorders we used and can fail to record the lower-amplitude parts of bat calls, such as feeding buzzes [42]. Since LED lights do not affect bat activity in any way that is different from LPS lights, replacing LPS lights with LED rather than metal halide lights will cause less change to bat activity around street lights.

We recorded few bats from the genera *Myotis*, *Plecotus* and *Rhinolophus*, probably because they avoid light when commuting and foraging; HPS and LED street lights showed similar effect sizes on reducing the number of passes of *Rhinolophus hipposideros* bats [21]. The low intensity echolocation calls of *Plecotus auritus* [42], the commonest species of *Plecotus* in Britain, will also have contributed to the paucity of data for this genus. The low numbers of *Myotis*, *Plecotus* and *Rhinolophus* bats we recorded is also likely to be attributable in part to the location of the study sites. Street lights are mostly in built-up locations, and so we worked in suburban areas where there were suitable habitats for bats. However, these are generally more open, less cluttered habitats, where slow-flying species of *Myotis*, *Plecotus* and *Rhinolophus* are less likely to occur [26,53], although we recorded a three-fold increase in *Myotis* spp. activity at site E following switch-over. While artificial lighting generally has a negative effect on *Myotis* species [21,27], this increase at the experimental site may be related to nearby swarming behaviour, which takes place in autumn, and would explain why there was such a large increase in bat activity despite a reduction in the number of feeding buzzes. When UV lights were erected in a desert in the USA, the insects attracted to the lamps were preyed on by a number of bats, including species of *Myotis* [54,55]; this may be due to differences in spectral properties and intensities between the UV lamps and the LED light studied here that emitted no UV.

If buzz ratio is a good proxy for insect activity, our results suggest that there is no difference in the absolute, but not necessarily relative, abundance of the groups of insects eaten by the species of bat we recorded [56] around LPS and LED street lights. While there have been no direct comparisons of insect activity around LPS and LED lights, there have between HPS and LED lights, although HPS lights have broader spectral emissions than LPS lights. However, the findings are conflicting. A study in New Zealand found that neutral 4000 K LED lights attracted 48% more insects than HPS lights [22], whereas a study in Germany with a mixture of cool (6500 K) and warm/neutral LEDs (3000/4100 K) found that more insects were attracted to HPS lights [57]. These differences may reflect differences in local insect communities, the habitats in which the two studies were carried out and/or because neither study was broad-scale.

The ecological impacts of artificial lighting are complex. More work is needed on how both bats and their insect prey, and other taxa, respond to different street lights before we can properly assess the ecological impacts of new lighting technologies, particularly LEDs, as these will soon be used worldwide and so have the potential for far-reaching ecological effects [58]. Due to our experimental design, there was some variation between sites in the power of the different light sources (10–107 watts) and correlated colour temperature (4000–5700 K) of the LED street lights we used. We were unable to control for this because the study was carried out in a “real-life” setting, and we had to use the lighting being installed by the local authority. However, we do not believe that colour temperature was a confounding variable because there is little difference in insect attraction between off-the-shelf LEDs with different colour temperatures (2700 K and 6000 K) [22]. To understand the effects of LED lighting on bats, and enable the results to be incorporated into lighting polices, it is important that future studies include all relevant information such as light source, output, spectral distribution, luminous flux and flicker rate [59], as well as data on habitat quality and environmental variables. It is also important that studies should involve multiple sites in different areas to avoid drawing conclusions based on local effects.

### Conservation perspective

From a conservation viewpoint, our results are encouraging because they suggest that the large-scale replacements of old lighting technologies by LED lights currently taking place in many parts of the UK, as well as in other countries [9], will not affect bat activity significantly differently from what currently occurs at LPS street lights. While there may be different impacts on other taxa, our data suggest that broad spectrum light sources such as LEDs will not necessarily have a greater ecological effect on bats than narrow spectrum lights [9].

However, it is important that these results are viewed alongside the wider impact of artificial lighting on bats. The majority of echolocation calls we recorded were from three species/groups of bats, which are typically considered as light-tolerant. There have been a number of studies showing the detrimental effects of lighting on roost emergence [60], commuting [31,61] and fitness [62] of a number of slow-flying bat species. Many of these are already vulnerable to habitat loss and urbanisation [63], and are further disadvantaged by the spread of artificial lighting.

## Conclusions

LED lights are widely perceived as being environmentally friendly because of their lower CO_2_ emissions. The results from this paired study also indicate that the switch-over from LPS to LED street lights did not affect the activity of bat species typically found in close proximity to street lights in suburban environments in the UK. The direction of change within a pair was consistent for eleven of the twelve sites and, as this experiment was carried out at a broad geographical scale, the switch-over from LPS to LED street lights is unlikely to have an effect on bat activity. From a conservation perspective this is a positive outcome as many existing street lights are being, or have already been, switched to LED in the UK and across the world. The lack of change in the number of feeding buzzes suggests that there was no significant change in the overall abundance around street lights of those insect groups eaten by bats, although more data are needed on individual insect groups, and how LEDs affect species interactions.

## Supporting Information

S1 Fig The arrangement of the SM3 bat detector and microphone on each of the lighting columns. (TIF)

S1 Table The number of passes and buzz ratios for total bat activity at the control and experimental lighting columns before and after the switch-over to LED lights. The buzz ratios are shown in brackets.
(DOCX)

S2 Table The number of bat passes and buzz ratios for *Pipistrellus pipistrellus* at the control and experimental lighting columns before and after the switch-over to LED lights. The buzz ratios are shown in brackets.
(DOCX)

S3 Table The number of bat passes for *Pipistrellus pygmaeus* at the control and experimental lighting columns before and after the switch-over to LED lights. (DOCX)

S4 Table The number of bat passes for *Nyctalus* spp. at the control and experimental lighting columns before and after the switch-over to LED lights. (DOCX)
