# Supplementary figures and images for: The Switch from Low-Pressure Sodium to Light Emitting Diodes Does Not Affect Bat Activity at Street Lights
Source: PLoS One. 2016 Mar 23;11(3):e0150884. doi: 10.1371/journal.pone.0150884 (PMC4805201; doi:10.1371/journal.pone.0150884)

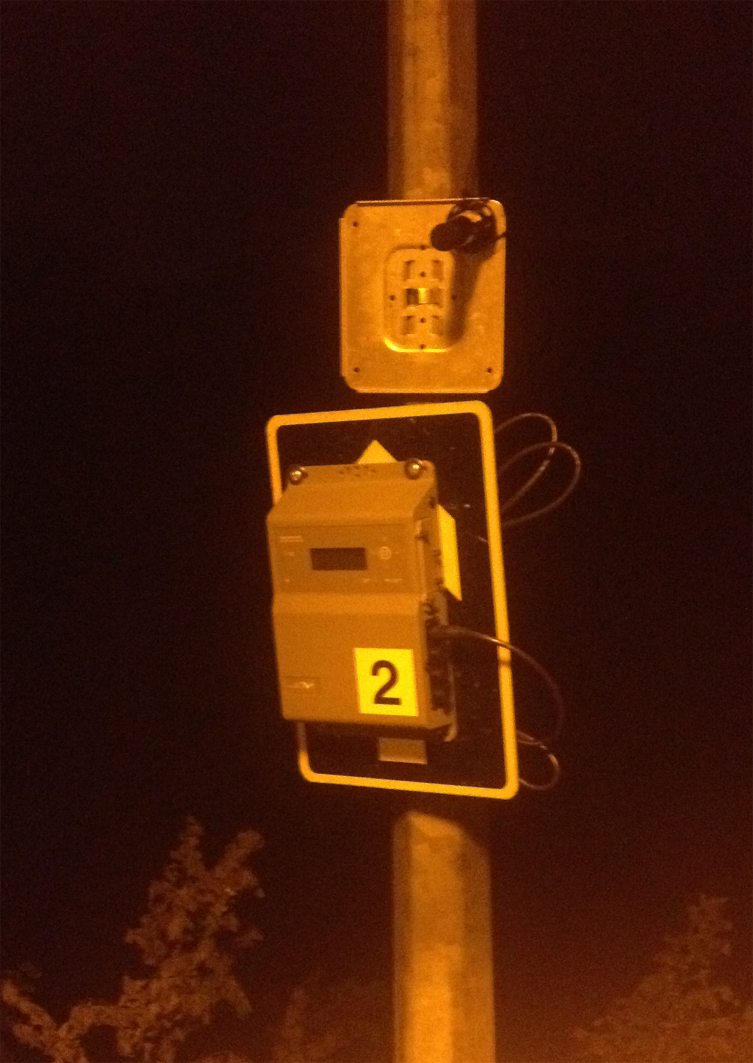

Supplement: S1 Fig — (TIF) [file pone.0150884.s001.tif]
