# Supplementary material for: The Switch from Low-Pressure Sodium to Light Emitting Diodes Does Not Affect Bat Activity at Street Lights
Source: PLoS One. 2016 Mar 23;11(3):e0150884. doi: 10.1371/journal.pone.0150884 (PMC4805201; doi:10.1371/journal.pone.0150884)
Supplement: S1 Table — The buzz ratios are shown in brackets. (DOCX) [file pone.0150884.s002.docx]

**S1 Table. The number of passes and buzz ratios for total bat activity at the control and experimental lighting columns before and after the switch-over to LED lights.** The buzz ratios are shown in brackets.

| **Site** | **Control** | | **Experimental** | |
| --- | --- | --- | --- | --- |
|  | **Before** | **After** | **Before** | **After** |
| A | 551 (0.17) | 350 (0.15) | 333 (0.07) | 351 (0.03) |
| B | 890 (0.16) | 179 (0.22) | 81 (0.15) | 36 (0.06) |
| C | 32 (0.06) | 167 (0.07) | 42 (0.05) | 62 (0.02) |
| D | 85 (0.14) | 24 (0.00) | 542 (0.07) | 180 (0.04) |
| E | 578 (0.10) | 1485 (0.28) | 421 (0.14) | 13,716 (0.05) |
| F | 578 (0.04) | 508 (0.07) | 599 (0.09) | 525 (0.14) |
| G | 341 (0.08) | 436 (0.11) | 172 (0.03) | 298 (0.06) |
| H | 473 (0.14) | 128 (0.02) | 656 (0.26) | 589 (0.03) |
| I | 2593 (0.15) | 522 (0.05) | 1963 (0.20) | 29 (0.00) |
| J | 485 (0.05) | 371 (0.01) | 1189 (0.29) | 1137 (0.13) |
| K | 273 (0.19) | 233 (0.11) | 1950 (0.16) | 236 (0.15) |
| L | 106 (0.10) | 302 (0.06) | 97 (0.02) | 230 (0.05) |
|  |  |  |  |  |
| Total | 6985 | 4705 | 8045 | 17,389 |
| Mean | 582.1 (0.12) | 392.1 (0.10) | 670.4 (0.13) | 1449.1 (0.06) |
| SD | 680.3 (0.05) | 377.1 (0.08) | 680.5 (0.09) | 3875.4 (0.05) |

Excluding site E, the total, mean and SD bat passes were:-

|  | **Control** | | **Experimental** | |
| --- | --- | --- | --- | --- |
|  | **Before** | **After** | **Before** | **After** |
| Total | 6407 | 3220 | 7624 | 3673 |
| Mean | 582.5 (0.12) | 292.7 (0.08) | 693.1 (0.13) | 333.9 (0.06) |
| SD | 713.5 (0.05) | 161.4 (0.06) | 709.0 (0.09) | 323.7 (0.05) |
