# Supplementary material for: The Switch from Low-Pressure Sodium to Light Emitting Diodes Does Not Affect Bat Activity at Street Lights
Source: PLoS One. 2016 Mar 23;11(3):e0150884. doi: 10.1371/journal.pone.0150884 (PMC4805201; doi:10.1371/journal.pone.0150884)
Supplement: S2 Table — The buzz ratios are shown in brackets. (DOCX) [file pone.0150884.s003.docx]

**S2 Table. The number of bat passes and buzz ratios for *Pipistrellus pipistrellus* at the control and experimental lighting columns before and after the switch-over to LED lights.** The buzz ratios are shown in brackets.

| **Site** | **Control** | | **Experimental** | |
| --- | --- | --- | --- | --- |
|  | **Before** | **After** | **Before** | **After** |
| A | 546 (0.17) | 332 (0.15) | 329 (0.06) | 343 (0.03) |
| B | 531 (0.16) | 179 (0.22) | 37 (0.10) | 27 (0.06) |
| C | 26 (0.06) | 153 (0.06) | 35 (0.05) | 44 (0.00) |
| D | 67 (0.14) | 19 (0.00) | 518 (0.07) | 172 (0.04) |
| E | 555 (0.10) | 1456 (0.28) | 378 (0.14) | 8867 (0.04) |
| F | 435 (0.03) | 269 (0.05) | 563 (0.09) | 495 (0.13) |
| G | 318 (0.08) | 380 (0.10) | 157 (0.03) | 243 (0.04) |
| H | 416 (0.13) | 27 (0.00) | 420 (0.22) | 18 (0.00) |
| I | 726 (0.05) | 86 (0.00) | 5 (0.00) | 3 (0.00) |
| J | 470 (0.05) | 369 (0.01) | 1171 (0.29) | 1123 (0.13) |
| K | 271 (0.19) | 218 (0.10) | 1941 (0.16) | 224 (0.15) |
| L | 59 (0.08) | 239 (0.06) | 40 (0.00) | 158 (0.04) |
|  |  |  |  |  |
| Total | 4420 | 3727 | 5594 | 11,717 |
| Mean | 368.3 (0.10) | 310.6 (0.09) | 466.2 (0.10) | 976.4 (0.06) |
| SD | 224.3 (0.05) | 380.7 (0.09) | 569.9 (0.09) | 2504.1 (0.05) |

Excluding site E, the total, mean and SD bat passes were:-

|  | **Control** | | **Experimental** | |
| --- | --- | --- | --- | --- |
|  | **Before** | **After** | **Before** | **After** |
| Total | 3865 | 2271 | 5216 | 2850 |
| Mean | 351.4 (0.10) | 206.5 (0.07) | 474.2 (0.10) | 259.1 (0.06) |
| SD | 227.1 (0.06) | 127.6 (0.07) | 597.0 (0.09) | 324.4 (0.06) |
