# Supplementary material for: The Switch from Low-Pressure Sodium to Light Emitting Diodes Does Not Affect Bat Activity at Street Lights
Source: PLoS One. 2016 Mar 23;11(3):e0150884. doi: 10.1371/journal.pone.0150884 (PMC4805201; doi:10.1371/journal.pone.0150884)
Supplement: S3 Table — (DOCX) [file pone.0150884.s004.docx]

**S3 Table. The number of bat passes for *Pipistrellus pygmaeus* at the control and experimental lighting columns before and after the switch-over to LED lights.**

| **Site** | **Control** | | **Experimental** | |
| --- | --- | --- | --- | --- |
|  | **Before** | **After** | **Before** | **After** |
| A | 3 | 13 | 4 | 5 |
| B | 5 | 0 | 1 | 2 |
| C | 0 | 1 | 7 | 5 |
| D | 8 | 5 | 6 | 1 |
| E | 1 | 1 | 6 | 1914 |
| F | 66 | 151 | 6 | 17 |
| G | 8 | 18 | 5 | 11 |
| H | 33 | 99 | 206 | 566 |
| I | 97 | 65 | 1 | 15 |
| J | 9 | 2 | 16 | 10 |
| K | 0 | 8 | 7 | 9 |
| L | 26 | 40 | 4 | 19 |
|  |  |  |  |  |
| Total | 256 | 403 | 269 | 2574 |
| Mean | 21.3 | 33.6 | 22.4 | 214.5 |
| SD | 30.6 | 48.1 | 57.9 | 558.6 |

Excluding site E, the total, mean and SD bat passes were:-

|  | **Control** | | **Experimental** | |
| --- | --- | --- | --- | --- |
|  | **Before** | **After** | **Before** | **After** |
| Total | 255 | 402 | 263 | 660 |
| Mean | 23.2 | 36.5 | 23.9 | 60.0 |
| SD | 31.4 | 49.3 | 60.5 | 167.9 |
