# Supplementary material for: The Switch from Low-Pressure Sodium to Light Emitting Diodes Does Not Affect Bat Activity at Street Lights
Source: PLoS One. 2016 Mar 23;11(3):e0150884. doi: 10.1371/journal.pone.0150884 (PMC4805201; doi:10.1371/journal.pone.0150884)
Supplement: S4 Table — (DOCX) [file pone.0150884.s005.docx]

**S4 Table. The number of bat passes for *Nyctalus* spp. at the control and experimental lighting columns before and after the switch-over to LED lights.**

| **Site** | **Control** | | **Experimental** | |
| --- | --- | --- | --- | --- |
|  | **Before** | **After** | **Before** | **After** |
| A | 1 | 4 | 0 | 1 |
| B | 134 | 0 | 41 | 7 |
| C | 0 | 11 | 0 | 13 |
| D | 10 | 0 | 11 | 6 |
| E | 19 | 26 | 26 | 60 |
| F | 61 | 85 | 28 | 12 |
| G | 14 | 38 | 6 | 40 |
| H | 7 | 0 | 14 | 0 |
| I | 1746 | 365 | 1888 | 11 |
| J | 2 | 0 | 0 | 0 |
| K | 1 | 5 | 1 | 1 |
| L | 18 | 20 | 45 | 51 |
|  |  |  |  |  |
| Total | 2013 | 554 | 2060 | 202 |
| Mean | 167.8 | 46.2 | 171.7 | 16.8 |
| SD | 498.5 | 103.4 | 540.7 | 21.2 |
